# Accurate staging of reproduction development in Cadenza wheat by non-destructive spike analysis

**DOI:** 10.1093/jxb/eraa156

**Published:** 2020-04-07

**Authors:** José Fernández-Gómez, Behzad Talle, Alison C Tidy, Zoe A Wilson

**Affiliations:** 1 School of Biosciences, University of Nottingham, Sutton Bonington Campus, Loughborough, Leicestershire, UK; 2 CSIRO Agriculture and Food, Australia

**Keywords:** Anther, barley, growth staging, last flag elongation, pollen development, Zadoks stage

## Abstract

Wheat is one of the most important crops in the world; however, loss of genetic variability and abiotic stress caused by variable climatic conditions threaten future productivity. Reproduction is critical for wheat yield; however, pollen development is amongst the developmental stages most sensitive to stresses such as heat, cold, or drought. A better understanding of how anther and pollen development is regulated is needed to help produce more resilient crops and ensure future yield increases. However, in cereals such as wheat, barley, and rice, flowers form within the developing pseudostem and therefore accurate staging of floral materials is extremely challenging. This makes detailed phenotypic and molecular analysis of floral development very difficult, particularly when limited plant material is available, for example with mutant or transgenic lines. Here we present an accurate approach to overcome this problem, by non-destructive staging of reproduction development in Cadenza, the widely used spring wheat research variety. This uses a double-scale system whereby anther and pollen development can be predicted in relation to spike size and spike position within the pseudostem. This system provides an easy, reproducible method that facilitates accurate sampling and analysis of floral materials, to enable anther and pollen developmental research.

## Teaser

We describe non-destructive staging of reproduction development in Cadenza, using a double-scale system whereby anther/pollen development can be predicted in relation to spike size and spike position within the pseudostem.

## Introduction

Wheat is one of the main staple foods worldwide and is the most abundant crop cultivated in the developing world, providing 20% of total dietary calories and proteins worldwide (Shiferaw *et al.*, 2013); however, loss of genetic variability due to domestication and abiotic stress caused by variable climatic conditions threaten future productivity (Smýkal, 2018). Wheat yearly production reached 14.1 Mt, with a total market value of US$2.63 billion in 2016 (Rabobank, 2016). However, increasing populations and a steady growth in wheat consumption alongside climatic variability means that wheat yields need to increase by an estimated 2.6% per annum to meet ongoing demand (Shiferaw *et al.*, 2013), which poses major challenges for breeders, researchers, and governments. In addition, the exhaustion of the effects which increased wheat yield during the green revolution (Whitford *et al.*, 2013) and new environmental policies mean that novel varieties capable of contributing to more sustainable and resilient crops are needed (Shiferaw *et al.*, 2013).

Multiple factors contribute to yield loss, from impoverishing of genetic variability due to intensive farming and domestication, and (a)biotic stresses. To help mitigate these adverse effects, introgression programmes are ongoing to introduce beneficial traits that were lost due to intensive farming and domestication, from ancestors into modern elite lines (Grewal *et al.*, 2018). In addition, new genomic tools have been developed to exploit the potential within the wheat genome. For example, genome sequencing, transformation, gene editing, and genomic tools are facilitating more ambitious research into wheat yield sensitivity to (a)biotic stresses (Dolferus *et al.*, 2011; Parish *et al.*, 2012; Liu *et al.*, 2015). Research carried out in Arabidopsis and rice has shown that stress can impair reproduction, particularly during meiosis, gametogenesis, and fertilization (Barnabas *et al*., 2008); for instance, drought (Ding *et al.*, 2018), heat, or cold stress (Zampieri *et al.*, 2017) cause abnormal pollen development, reducing fertility (Shimono *et al.*, 2016). The impact of stress damage is also particularly dependent on the stage when it occurs and the species. For instance, stress during pollen development in rice, when the tapetum activity is high during the transition between tetrads and early microspore release, produces major damage (Oliver *et al.*, 2005; Chaturvedi *et al.*, 2017).

However, research on flower development, fertility regulation, and associated sensitivity to environmental stress in wheat is significantly limited by the accurate staging of anther and pollen development. From early reproductive development until spike emergence, floral development occurs within the pesudostem, and thus is not visible without destructive analysis. This means that it is extremely difficult to accurately stage and collect floral samples for phenotypic or molecular analysis without damaging the plant; this is particularly important when limited plant resources are available, for example for mutant or transgenic lines. It is therefore necessary to develop staging systems based on overall plant morphology and development that can be linked to anther and pollen development, instead of using samples collected based on plant age (Simmons *et al.*, 2006; Ninkovic and Åhman, 2009). Non-destructive prediction methods are preferable to conventional anther or floret measurements that require plant dissection (Gómez and Wilson, 2012).

The prediction of anther and pollen development has been approached in different ways; generally anther and pollen stages appear to be tightly linked to spike and anther size in barley and wheat (Waddington *et al.*, 1983; Kirby, 1988). Waddington *et al.* (1983) developed a scoring system for barley and wheat that correlates spike development with anther length. In addition, other studies correlate floret size and anther length with stages of anther development in rice (Raghavan, 1988). Recently a study of four Australian wheat varieties has shown that spikelet and anther size can be used to accurately predict anther stages (Browne *et al.*, 2018). However, these systems require the dissection of plants to measure the parameters used to predict the anther and pollen development stages and therefore are not suitable for ongoing developmental analyses. Gomez and Wilson (2012) previously developed a non-destructive staging system in barley based upon the Zadok stages, that combined node number, last flag elongation (LFE), and ear position within the plant to predict spike size in barley, which was then linked to anther and pollen development stages (Gómez and Wilson, 2012). In addition, a non-destructive method of measuring the auricula distance (AD; distance between the auricles of the flag leaf and the penultimate leaf) has been used in wheat and rice to predict the young microspore stage (Morgan, 1980; Oliver *et al.*, 2005; Ji *et al.*, 2010); however, this approach is varietal specific and has limitations in its ability to predict all anther stages.

Here we present a non-destructive anther and pollen development staging system in the wheat variety Cadenza. Cadenza is a widely used research variety that was the genotype selected to generate the wheat Tilling Exon Capture Population (King *et al.*, 2015). This publicly available population contains thousands of mutant lines, which have annotated gene mutations that are available for public use (https://www.seedstor.ac.uk/shopping-cart-tilling.php). The development of this prediction system will enable accurate analysis of anther and pollen development which, combined with the availability of this mutant population, will allow ambitious research programmes on reproductive development.

## Materials and methods

### Plant materials

Spring wheat variety Cadenza was grown under controlled conditions of 18 ºC/14 ºC and a 16 h photoperiod [80% relative humidity, 500 µmol m^–2^ s^–1^ metal halide lamps (HQI) supplemented with tungsten bulbs]. Seeds were sown in 12-well (80 ml each) pots using John Innes No. 3 compost (https://www.gardenhealth.com/j-arthur-bowers-john-innes-3-compost). After 2–3 weeks (Zadoks stage 11–12: one shoot with one or two unfolded leaves), plants were transferred to 5 litre pots containing Levington CNS compost (90N 46P 150K) with three plants per pot. Plants were fed once after being transferred to a 5 litre pot with Osmocote Exact Tablets (5 g; 16-9-12 + 2MgO+TE; http://icl-sf.com).

### Morphological analysis

**Fig. 1.**
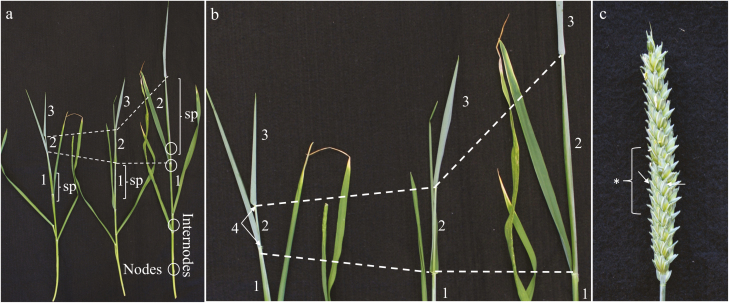
Cadenza last flag sheath development. (a) Three tillers at different stages of development showing different last flag sheath extension; sp indicates the spike position within the pseudostem. White circles show the nodes. (b) Close up view of last flag and last sheath at three different elongation stages. (c) Close up image of wheat spike indicating the middle area used for sample collection for sectioning and qRT–PCR. 1, PLS (previous last sheath); 2, LS (last sheath); 3, last flag; 4, auricle; sp, spike position; *Spike middle region for sample collection; White arrows indicates spikelet 1 and 2 position, which were the only spikelets used for collection.

Cadenza morphological development was studied from the onset of the elongation stage to anther anthesis to gain a better understanding of Cadenza development and to establish clearly recognizable features that could be related to anther development. Zadoks decimal code was initially used to identify key developmental points (Zadoks *et al.*, 1974); however, new time points were used to establish a correlation between morphological and reproductive development. Five plants, with four tillers per plant, were used. Material used for data collection was restricted to tillers that were already within the elongation stage when the main shoot was around the booting stage. The florets analysed were collected from spikelet position 1 and 2, and always in the central positions within the spike (Fig. 1c). Data were collected every 3 d and consisted of: total height, number of nodes, internode elongation, flag leaf emergence (shoot axis elongation), spike position within the pseudostem, spike emergence, peduncule elongation after total spike emergence, and spike size (Fig. 1).

In addition, a separate replicate set of plants growing under the same conditions were used for spike size analysis. All the data parameters were collected; however, these plants were dissected to measure the spike and to collect anther samples for sectioning and quantitative reverse transcription–PCR (qRT–PCR). Further generations of material were also subsequently grown for analysis and confirmation of the staging system.

### Histological analysis of anther development

Florets from the middle zone of the spike were collected from different spike developmental stages. Floral buds from different stages were fixed overnight at 4 ºC in 4% (v/v) paraformaldehyde. Tissues were washed twice (30 min each) with 1× phosphate-buffered saline (PBS). Fixed panicles were immediately dehydrated with ethanol [30–100% (v/v)] prior to embedding in Spurr resin (TAAB Laboratories Equipment, Ltd). After 100% ethanol, samples had a mixture of 100% ethanol and propylene oxide (Sigma) 1:1 added, which was replaced by 100% propylene oxide after 20 min. As a pre-infiltration step, samples were mixed with propylene oxide/Spurr resin (1:1) for 1.5 h, and the solution was then replaced with 100% Spurr resin and kept at 4 ºC overnight. After this, samples were embedded in moulds using Spurr resin and incubated at 70–75 °C for 10–12 h. Ultrathin sections (0.5 µm) were produced using an Leica EM UC6 ultramicrotome. Sections were stained with 0.25% (w/v in 1% sodium borate) toluidine blue prior to imaging.

### Anther-specific gene expression

Due to the high specificity of some of the genes expressed in the anther development network, expression analysis was used as an additional approach to confirm the correlation between the samples collected and the expected stages. Samples were collected using spike size/position scale covering all spike stages. RNA was purified using RNeasy spin columns (Qiagen). First-strand cDNAs were synthesized from 1.5 µg of total RNA using Superscript III reverse transcriptase (Invitrogen) and an oligo(dT) primer (Invitrogen) according to the manufacturer’s instructions. Three anther-specific genes were used for staging, *TaDYT1*, *TaMS1*, and *TaMYB26*. TaDYT1 and TaMS1 are essential tapetum-specific transcription factors, whereas TaMYB26 plays a critical role in anther dehiscence.

### X-ray micro computed tomography (µCT) scanning

X-ray µCT scanning of wheat plants was performed using the v|tome|x M 240 kV X-ray µCT scanner (GE Sensing & Inspection Technologies GmbH, Wunstorf, Germany). Scanning conditions were 80 kV voltage, 250 current, 250 timing, 32 average, 0 skips, 1×1 binning, and 4.0 sensitivity. A single radiograph consisting of 32 integrated images was taken to limit the X-ray dose to the plant to 10 s per radiograph (total exposure 15 min over all scans). Spikes and nodes were easily identifiable by this method, and close-up images of each spike being monitored were taken, as well as an overall picture. Three plants were taken from early flowering, and individual tillers were identified and monitored over 2 weeks (days 0, 7, and 14) to monitor spike growth and position (Tracy *et al.*, 2017).

## Results

### Plant morphology development in wheat variety Cadenza

**Fig. 2.**
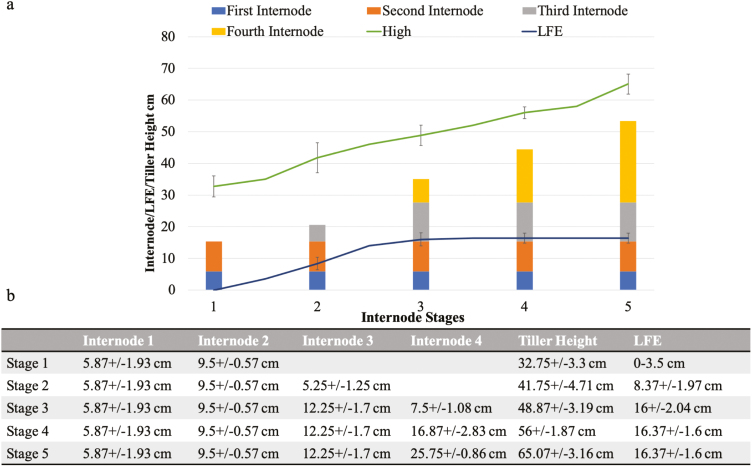
Cadenza morphological development. (a) Internode elongation stages were determined by measuring internode elongation every 3 d. Tiller height (green line) and last flag elongation (LFE; blue line) were also measured. (b) Five internode stages were created, starting from two fully elongated internodes (internode stage 1), until four fully elongated internodes (internode stage 5). Tiller height increased steadily from stage 1 to 5, reaching a maximum of 65.1±3.2 cm. LFE appeared between stages 1 and 2 and reached a maximum elongation at stage 3.

**Fig. 3.**
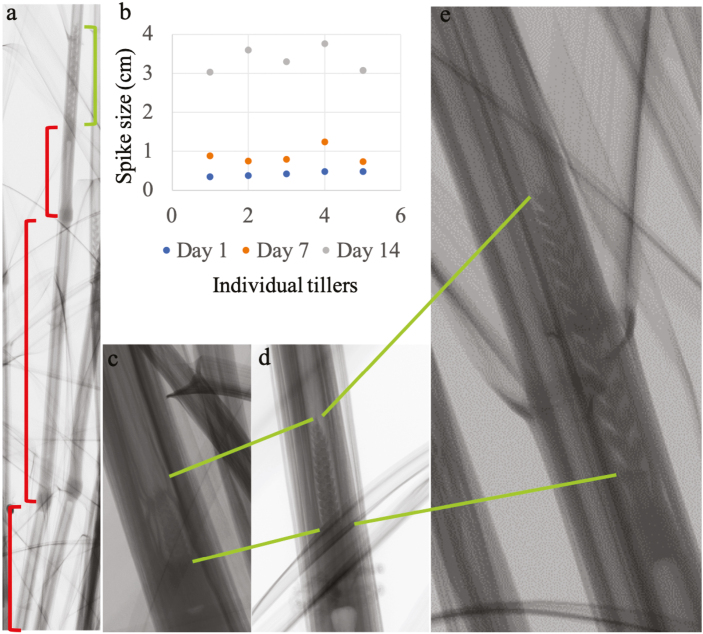
Cadenza internode and spike size development. (a) Internode elongation contribution to the spike upward movement (red brackets). (b) Spike measurement started on day 48 after sowing (Zadock stage 30; spikes were ~0.5 cm long). (a, c–e) µCT analysis of Cadenza spikelets. Spike size increase was observed over the following 14 d; spikes were up to 1.2 cm and >3 cm after 7 d and 14 d, respectively. (c–e) Spike observed on (c) day 1, (d) day 7, and (e) day 14.

**Fig. 4.**
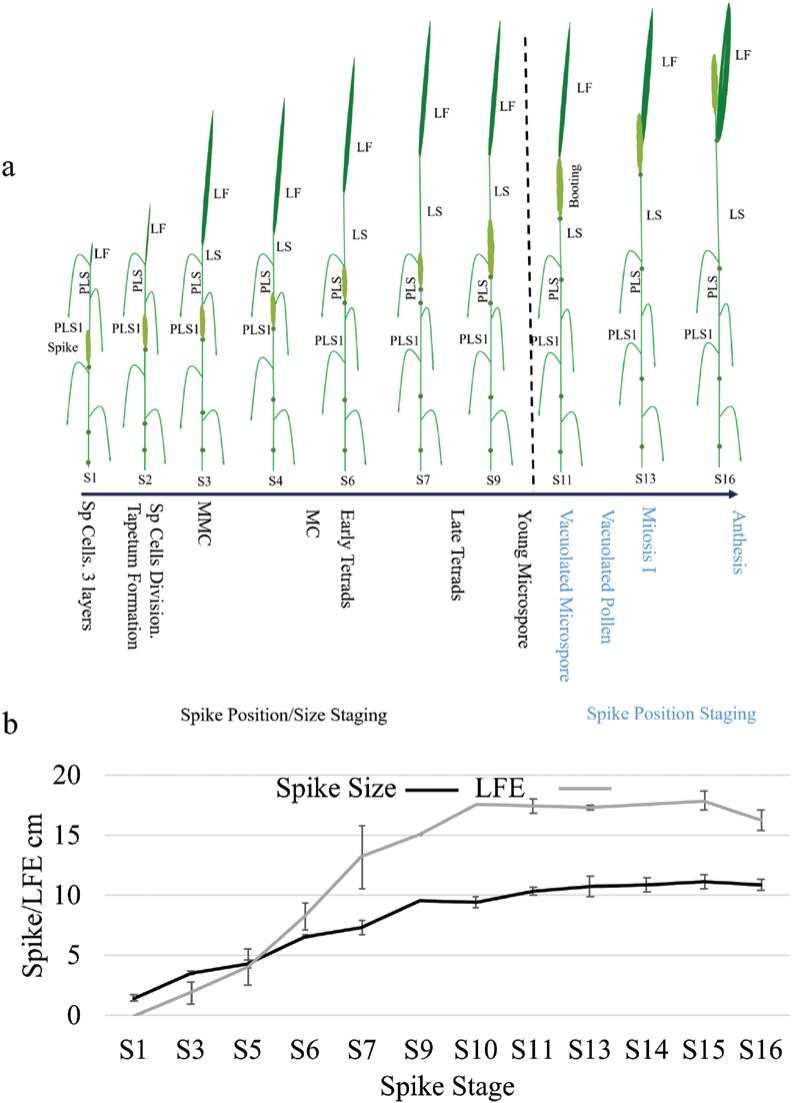
Cadenza spike position stages and spike size/last flag elongation (LFE) progression. (a) Spike position was divided into 16 stages, from early development, just before the spike enters the ‘prior to previous last sheath’ (PLS1) spike stage 1, until the spike is completely emerged, the peduncule is elongated, and anthers enter anthesis (spike stage 16). These 16 stages were linked to spike position within the pseudostem. (b) Spike stage/spike size correlation. Spike position and spike size showed a close correlation from stage 1 until stages 9–10. From stages 9–10, the spike ceased growing and remained unchanged.

**Fig. 5.**
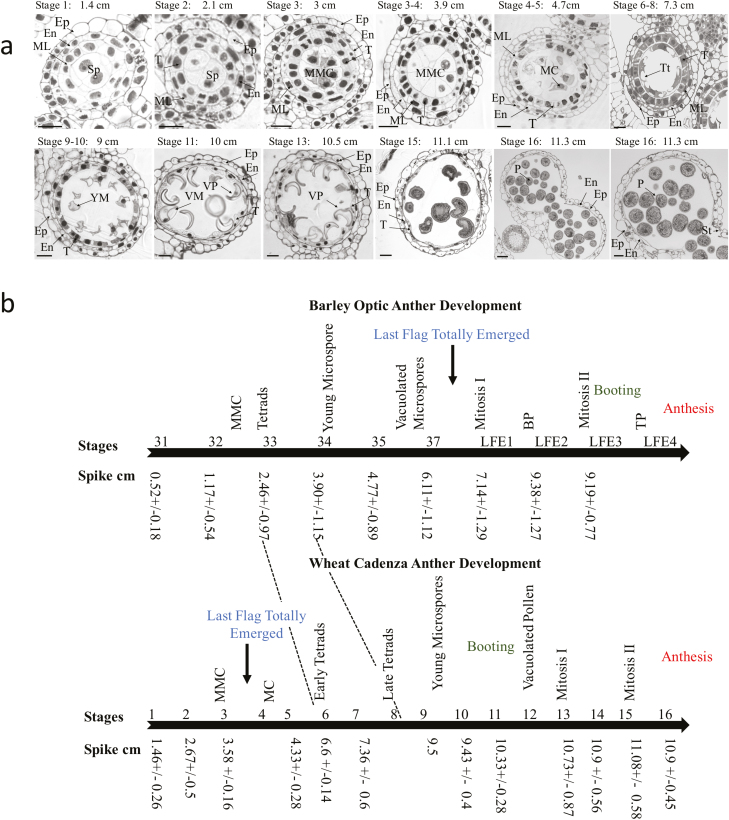
Developmentally staged Cadenza anther transverse sections. (a) Stages 1–16 of Cadenza anther development. (1) Sporogenous stage; three anther layers are visible (arrow; Ep, En, and ML) and sporogenous cells occupy the central region of the locule. (2) Sporogenous cells divide. Three anther layers are clearly visible; start of tapetum formation (T). (3) Microspore mother cells (MMCs) are evident. Four anther layers are visible (Ep, En, ML, and T). (3–4) MMCs divide, giving rise to (4–5) meiotic cells (MCs). (6–8) Tetrads (Tt) appear concentrically in contact with tapetum cells; the central locule appears empty and the middle layer (ML) becomes crushed. (9–10) Young microspores (YMs) released from tetrads; the ML is completely degenerated. (11 and 13) Tapetum starts degenerating. Vacuolated microspores (VMs) and vacuolated pollen (VP) formed. (15) Pollen mitosis I; the tapetum is still present. (16) Anther dehiscence. The septum has degenerated leaving two anther locules; the stomium still remains intact. (b) Comparison between barley (Optic) and wheat (Cadenza) anther and pollen development. Sp, sporogenous tissue; Ep, epidermis; En, endothecium; ML, middle layer; T, tapetum; L, lacunae; StR, stomium region; MMC, microspore mother cells; Tt, tetrads; YM, young microspores; VM, vacuolate microspores; VP, vacuolate pollen; MC, meiotic cell; St, stomium. Scale bar=0.02 mm.

Plant development was studied to establish a correlation between external morphology and anther developmental stages for the wheat cultivar Cadenza (Fig. 1). In order to gain knowledge about Cadenza’s external morphological development, the emergence of nodes and the internode elongation were noted and total tiller height and last flag elongation were measured (Fig. 2). In addition, spike position within the pseudostem and its relationship to spike size was determined (Figs 3, 4; Table 1). Finally, a system that relates spike size/positioning within the plant and anther staging was established (Figs 4, 5; Table 1).

**Table 1.** Spike position staging system

Spike stage	Description	Spike size	Anther stage
Stage 1	Spike starts PLS1	1.46±0.26 cm	Four lobes formed. Sporogenous cells present. Three cell layers surrounding anther locule.
Stage 2	Spike middle PLS1	2.67±0.5 cm	Sporogenous cells divide. Tapetum being formed.
Stage 3	Spike top PLS1, about to enter PLS	3.58±0.16 cm	Microspore mother cells.
Stage 4	Spike one-quarter within PLS		Microspore mother cells to meiotic cells.
Stage 5	Spike all in PLS	4.33±0.28 cm	
Stage 6	Spike all in PLS about to enter LS	6.6±0.14 cm	Tetrads.
Stage 7	Spike one-quarter within LS	7.36±0.60 cm	
Stage 8	Spike half within LS		
Stage 9	Spike three-quarters within LS	9.5 cm	Young microspore release. Tapetum commences degeneration.
Stage 10	Spike all within LS	9.43±0.4 cm	
Stage 11	Spike all within LS. Booting	10.33±0.28 cm	Vacuolated microspore–vacuolated pollen. Tapetum visibly degenerating.
Stage 12	Spike one-quarter out		
Stage 13	Spike half out	10.73±0.87 cm	Vacuolated pollen. Mitosis I starts.
Stage 14	Spike three-quarters out	10.9±0.56 cm	Mitosis I.
Stage 15	Spike all out 0–3 cm	11.08±0.58 cm	Mitosis II.
Stage 16	Spike all out elongated >3 cm	10.9±0.45 cm	Anthesis.

Spike size/position correlates accurately with anther development stages. From stage 1 to 9, spike continuously grew in size; however, from stages 9–10, spike ceased growing. LS, last sheath; PLS, previous last sheath; PLS1, prior to previous last sheath.

### Internode and last flag leaf (LFE) elongation development

Nodes and internode elongation, total tiller height, and LFE data were collected every 3 d from the two detectable nodes until spikes were out and the peduncule extended (Fig. 2). Data collected permitted the creation of five internode stages based on node visibility and internode elongation (Figs 2, 3a (red bracketed regions), which were also confirmed by µCT analysis (Fig. 3). Three main stages (1, 3, and 5), and two intermediate stages (2 and 4) were created encompasing all points of reproductive development (Fig. 2). At internode stage 1, internodes 1 and 2 were fully elongated, whereas at stage 3, the first three internodes were fully elongated, plus the fourth was elongated only a quarter of its expected maximum (Fig. 2). Finally, by internode stage 5, all internodes (1–4) had reached maximum elongation (Fig. 2). To increase accuracy, two intermediate stages were included which recorded the production of internodes between the main stages (Fig. 2). At stage 2, the first two internodes had elongated fully and the third only to half of its expected maximum (Fig. 2), whilst at stage 4, the first three internodes were fully elongated and the fourth had elongated to three-quarters of its expected maximum (Fig. 2).

Tiller height was measured from the plant base, until the last visible auricle (Fig. 1b). Height increase progressed steadily from 32.75 cm at internode stage 1, until 65.07 cm at internode stage 5 (Fig. 2). LFE was measured from its first appearance between internode stages 1 and 2, until the end of plant development at internode stage 5 (Figs 1a, 2). LFE showed a constant elongation until internode stage 3 (Fig. 2a), after which it remained unaltered until the end of tiller development (Fig. 2).

### Spike upward movement and development within the spike

From the start of the reproductive stage (first node detectable separated by >1 cm from the ground) (Zadoks *et al.*, 1974), the spike starts an upward movement driven by internode elongation that continues until the spike is completely emerged and the peduncle stops elongating (Fig. 4a). This spike upward movement was divided into 16 identifiable points (spike stages) (Fig. 4a; Table 1). These spike stages started from the point at which the spike was completely in PLS1 (prior to previous last sheath) (Fig. 4a, spike stage 1). Five more stages (2–6) were established describing the spike upward movement between PLS1 and PLS (previous last sheath) (Fig. 4a). Stages 2 and 3 reflect the transition within PLS1, entering into PLS at stage 4. Spike upward movement within PLS continued between stages 5 and 6, just before entering the last sheath (LS) stage. From stage 6, five more stages were established describing the spike’s movement within the LS (stages 7–11) (Fig. 4a). From stage 7 (spike one-quarter within the LS), the spike moves upward to reach half way (stage 8), three-quarters (stage 9), and all within (stage 10) the LS. The internode extension subsequently pushes the spike further up within the leaf sheath, bringing the spike to the top of the leaf sheath immediately before emergence (stage 11, booting) (Fig. 4a). From this point, and continuing until spike emergence, five more stages were characterized: spike one-quarter emerged (stage 12), half way (stage 13) and three-quarters out (stage 14), spike fully out (peduncule elongated between 0–3 cm, stage 15), and finally spike fully out with peduncule elongated >3 cm (Fig. 4a; Table 1).

Spike stage correlation with spike size was determined by measuring spikes collected using our spike staging system (Fig. 4b; Table 1). Spikes showed a continuous growth from Zadok stage 30, spike ~0.5 cm (sample day 1, Fig. 3b), and could be easily linked to spike position from spike stage 1 (1.5±0.3 cm) until stages 9–10 (9.5±0.4 cm) (Fig. 4b; Table 1). After stage 9, the spike reached its maximum size (10–11 cm), remaining unchanged until the end of development at stage 16 (Fig. 4b).

### The combination of spike size and its position within the pseudostem can be used to predict anther development

To establish a correlation between spike size/position and anther development, spike staging was used to collect different spike samples for ultra-thin microscopy. Due to the spike ceasing to grow from stage 9 onward (Fig. 4b), spike size prediction was used to collect samples from stage 1 to stage 9, whereas spike position was used between stages 10 and 16 (Figs 4, 5).

Anther transverse sections showed a close relationship between spike size/position and anther stage (Figs 4, 5; Table 1). Anthers collected at stage 1 (spike ~1.4 cm) showed three anther layers (epidermis, endothecium, and middle layer) and sporogenous cells occupying the central locule (Fig. 5a). Between stages 2 and 3, the sporogenous cells continued dividing and the tapetum became apparent (Fig. 5, spikes 2.1–3 cm). At stage 3–4, all four anther layers were clearly visible and microspore mother cells (MMCs) were formed (Fig. 5, spike 3–3.9 cm). MMCs entered into meiosis at stage 4 (Fig. 5, spike 4.7 cm) and by stage 6 early tetrads were visible (stage 6, ear 6.2 cm, Table 1). The tetrad stage continued until stages 7–8 (Fig. 5, spike 7.3 cm). Between stages 6 and 8, the middle layer started to disappear (Fig. 5). At stages 9–10, young microspores were released from tetrads, with no middle layer observed (Fig. 5; spike 9 cm). Vacuolated microspores and pollen grains were visible at stages 11–12, and the tapetum started to degenerate between stages 10 and 11 coinciding with the release of microspores and the formation of vacuolated microspores (Fig. 5). Pollen mitosis I occurred at stages 13–14 (Fig. 5); the tapetum layer was still partly present. Stage 16 showed septum lysis, leaving two anther locules prior to anther dehiscence (Fig. 5).

This combined staging approach allowed the identification of critical anther development stages using easily identifiable spike size and positioning within the pseudostem stages (Fig. 4; Table 1). The system was divided into two parts due to the spike ceasing to grow after stage 9. From stages 1 to 9, spike size, as determined using spike position, was used to predict anther development progression. This allowed the identification of critical stages for male fertility such as tapetum formation (stages 2–3), meiosis (stages 4–5), and tetrad formation (stages 6–8) (Figs 4, 5). From stages 10 to 16, samples were selected only based on spike position, identifying stages from young microspore release (stage 9) until anthesis (stage 16) (Figs 4, 5).

### Anther- and pollen-specific expression corresponds to expected developmental staging

A non-destructive accurate anther and pollen development staging system is critical to study male fertility, and to perform gene expression profiles, mutant expression analysis, or RNA sequencing (RNAseq) sample collection. Therefore, sampling accuracy is vital. To test the accuracy of our staging method (based upon spike size and position) in predicting anther development stages, three highly anther-specific transcription factors, MS1, MYB26, and DYT1, were selected for gene expression analysis. These three transcription factors have been characterized in Arabidopsis, rice, and barley, and are involved in pollen formation (MS1 and DYT1) and anther dehiscence (MYB26). The three wheat orthologues were found using barley equivalent sequences by comparative analysis against the wheat database (https://plants.ensembl.org/Triticum_aestivum/Info/Index) (see Supplementary Fig. S1 at *JXB* online). Expression was analysed using primers that amplify the three homeologues simultaneously (Supplementary Table 1).

**Fig. 6.**
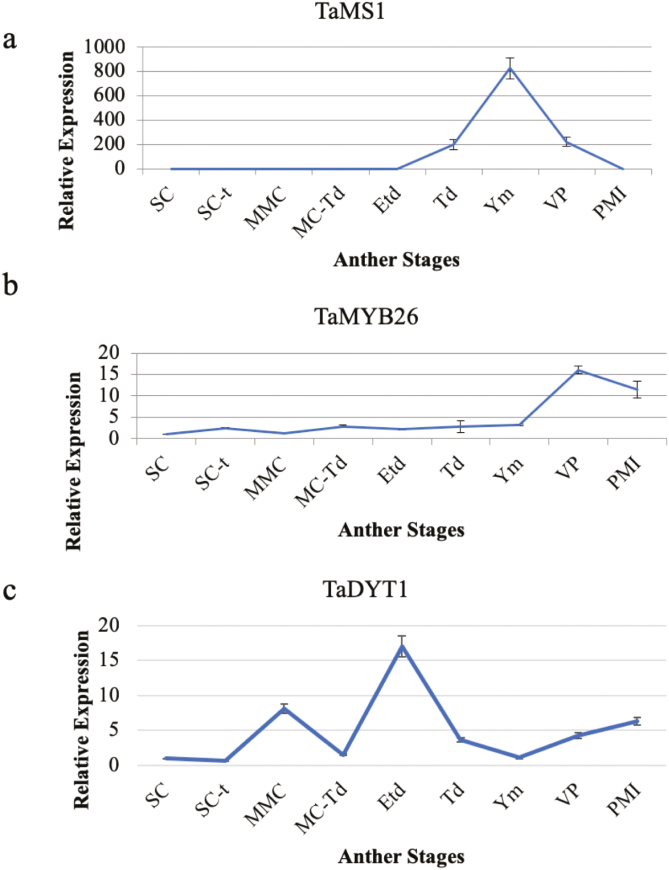
Expression patterns of anther-specific transcription factors by qRT–PCR analysis. (a) TaMS1, (b) TaMYB26, and (c) TaDYT1. SC, sporogenous cells; Sc-t, sporogenous cells–tapetum generation; MMC, microspore mother cells; MC, meiotic cells; Td, tetrads; Ym, young microspores; VP, vacuolated pollen; MtI, mitosis I.

*MS1* in Arabidopsis, rice, and barley is expressed in the tapetum just before microspore release as the callose surrounding the tetrad starts to break down until the free microspore stage (Wilson *et al.*, 2001; Li *et al.*, 2011; Fernandez Gomez and Wilson, 2014). MYB26, a transcription factor that regulates secondary thickening in the endothecium and is essential for anther dehiscence, is expressed just before mitosis I until the bicellular pollen stage (Yang *et al.*, 2007). Finally *UDT1*, the Arabidopsis *DYT1* orthologue in rice, shows a bimodal expression pattern with two expression peaks, at meiosis and at early tetrad stage (Jung *et al.*, 2005). The expression patterns of the putative wheat orthologues showed similar profiles to those observed in Arabidopsis, rice, and barley. *TaMS1* expression was observed in anthers between early tetrad to vacuolated pollen stage, reaching maximum expression at early microspore release (Fig. 6a). Furthermore, *TaMYB26* showed maximum expression between vacuolated pollen and mitosis I stage (Fig. 6b). *TaDYT1* showed low expression from sporogenous cells (SC) to sporogenous cells–tapetum generation (SCt) stages, increasing at the free microspore stage (Ms), then decreasing again at the microspore–tapetum transition stage (MC-Tt). From MC-Tt, expression reaches its peak at early tetrad stages (ETd), decreasing again around young microspores (Ym) and increasing slightly at mitosis I (Fig. 6c). These results confirm the accuracy of the non-destructive staging system for collection of stage-specific samples, enabling higher precision in sample collection which is needed for detailed molecular and physiological analysis of pollen development.

## Discussion

### Cadenza development

Predicting anther and pollen development stages is key to investigating plant fertility, the sensitivity of this critical trait to environmental stress, and the potential generation of hybrid seeds. Facilitating effective sample collection can also contribute to more homogeneous sampling sets in experiments such as RNAseq, or expression analysis, that will facilitate the identification and characterization of genes of interest and their manipulation.

The wheat cultivar Cadenza has been extensively used as a research tool, and recently a multinational effort has generated an exon capture population that is contributing to accelerate wheat research (King *et al.*, 2015). Therefore, this variety is a good model for developmental studies, and the generation of a system for accurate anther staging will capitalize on the existent mutant population and contribute to male fertility gene characterization. Physiological measurements, destructive staging using sectioning, and non-destructive staging were all used to generate combined data on the progression of Cadenza flower development. X-ray μCT contributed to the analysis of spikes at a very early stage in development (spike <1 cm; undifferentiated tissue) to assist in the developmental assessment and in additional validation of our final staging system (Fig. 3).

Wheat anther staging has been studied in detail by Mizelle *et al.* (1989); however, no relationship between external development and anther development was established (Mizelle *et al.*, 1989). Nonetheless, attempts to establish relationships between spikelet and anther sizes have proven very accurate in predicting these stages (Waddington *et al.*, 1983; Kirby, 1988; Browne *et al.*, 2018); however, these systems require plant dissection and therefore destruction of the plant materials.

Non-destructive methods based on auricle distance (AD) have been used in rice (Oliver *et al.*, 2005) and wheat (Ji *et al.*, 2010) to predict the young microspore stage in cold and drought stress treatments. However, the relationship between AD and anther stages in wheat proved highly varietal specific (Browne *et al.*, 2018). This was confirmed in Cadenza where LFE (the name used instead of AD in this study) proved to be inadequate to predict all anther stages due to the early cessation of elongation observed in the last flag around spike stages 9–10 (Fig. 2).

Parallelism between pseudostem and spike development has been described previously during wheat elongation stages (Kirby, 1988; Reynolds *et al.*, 2009), and a correlation was made between increase in spike size and floret development scores (Waddington *et al.*, 1983). However, establishing a relationship between external morphological changes, spike size, and anther stages is difficult due to differences in varieties and by the ongoing spike development within the pseudostem. In barley, this relationship between external characters and spike sizes/anther stages was successfully established (Gómez and Wilson, 2012) by modification of the traditional Zadok developmental scale (Zadoks *et al.*, 1974) and the introduction of additional marker stages. Clear prediction of spike size was possible for most stages of barley floral development; however, as the spike stopped growing just after the last flag started to elongate, a combination of last flag elongation and spike position within the plant was adopted to increase the accuracy of stage identification (Gómez and Wilson, 2012). As in barley, Cadenza’s spike reached a maximum size around spike stages 9–10 (Fig. 4b), at the point when the spike is entering the last sheath and microspores have been released from tetrads (Figs 5, 6). After this point, prediction of stage based on spike size alone cannot discriminate between these critical anther developmental stages (Figs 4, 5; Table 1).

### Anther stage prediction system

Internode elongation stages were initially targeted as the principal morphological parameter to link with anther development (Fig. 2a); however, this meant that predictions of internode maximum elongation were needed to establish the internode stages, which proved to be complex. Nonetheless, internode stages contributed to increasing the knowledge regarding Cadenza development and morphology. This knowledge facilitated the generation of a spike position staging which proved highly consistent and easy to follow, as stated here and in previous investigations (Kirby, 1988; Reynolds *et al.*, 2009; Gómez and Wilson, 2012) (Fig. 4; Table 1).

Spike size and its correlation with spike position resulted in a very accurate prediction of anther development stages (Table 1). As in barley (Gómez and Wilson, 2012), wheat spike size was valuable to predict anther and pollen development, at least until stages where the spike ceased growing (spike stage 9; Fig. 4; Table 1). From this point, anther staging prediction was continued by using the spike position within the leaf sheath which showed a clear correlation with anther stages until anther dehiscence (stages 10–16; Figs 4, 5; Table 1). Therefore, this double scale facilitated the identification of the stages from the onset of plant reproduction until anthesis (Figs 4, 5); this provides a tool for accurate sample collection aimed at analysing gene expression and developmental characterization (Fig. 6; Table 1). This staging system was confirmed using µCT and qRT–PCR expression analysis of key anther-specific genes; this validated the staging system, but is not required for subsequent accurate analysis of staging of materials.

Similar anther and pollen development progress was observed in wheat and barley (Fig. 5b); however, differences were observed in their timing and duration. For instance, in barley, stages such as tetrads and young microspore release occurred earlier in plant development, before all nodes were detected and extended (barley stages 33–34), and spikes were between 2.5 cm and 4.5 cm (Gómez and Wilson, 2012). In addition, these important stages all occurred before the last flag had started elongating (Gómez and Wilson, 2012) (Fig. 5b). On the other hand, in wheat, the early tetrad stage occurred at spike stages 5–6 when spikes were >6 cm long and the spike was about to enter the last flag sheath, which is already elongated to >8 cm (Fig. 4; Table 1). Moreover, it was observed that the wheat tetrad stage lasted longer than in barley (Fig. 5b). In wheat, meiosis was observed at spike stages 4–5 (spike ~4.5 cm), with tetrads evident soon after (stages 5–6, spike ~6.2 cm) (Table 1), and lasting until stage 8 (spike 7–8 cm). These stages corresponded to spike stages just before entering the last sheath (stage 6) until the spike was half within the spike (stage 8) (Fig. 4), whereas the tetrad stage in barley was much quicker, lasting from spikes of 3–4 cm (Gómez and Wilson, 2012), or Zadoks stages 33–33.5 (Zadoks *et al.*, 1974) (Fig. 5b). Environmental conditions and genotype are likely to impact upon developmental progression, and slight variation has been seen between different wheat genotypes (data not shown). Our floral staging system was developed for Cadenza grown under controlled-environment conditions since it is extensively used as a model for wheat molecular genetic analysis; nevertheless, this staging system provides a basis for direct application to other wheat genotypes and growth conditions, and can be readily correlated to other wheat genotypes/growth conditions.

This system of using spike size and position to determine anther development stage was further validated by expression analysis of three anther-/stage-specific transcription factors, *TaMS1*, *TaMYB26*, and *TaDYT1* (Wilson *et al.*, 2001; Jung *et al.*, 2005; Yang *et al.*, 2007). Samples collected using the spike size/position staging system were analysed by qRT–PCR (Fig. 6) to confirm the stages. Expression analysis for the three wheat genes were similar to the expression of their orthologues in Arabidopsis (Wilson *et al.*, 2001; Yang *et al.*, 2007), rice (Jung *et al.*, 2005; Li *et al.*, 2011), and barley (Fernandez Gomez and Wilson, 2014). Some differences were observed in the MYB26 expression pattern that is restricted to early mitosis I until bicellular pollen in Arabidopsis (Yang *et al.*, 2007), whereas in wheat expression seems to be more widespread throughout development (Fig. 6b). This confirms the accuracy of this staging system to predict anther development by a non-destructive method in Cadenza. This method, although focused upon Cadenza growing under control conditions, provides a valuable tool for anther and pollen stage prediction that will contribute to increased understanding of male fertility by enabling correct sampling of materials to facilitate gene expression analysis, RNAseq sampling, and gene characterization.

## Supplementary data

Supplementary data are available at *JXB* online.

Fig. S1. Interspecies similarities between the three anther transcription factors used for expression analysis.

**Table S1.** Primers used for qRT–PCR analysis of wheat anther-specific transcription factors.

eraa156_suppl_supplementary_table_S1_figure_S1
