# Supplementary material for: Accurate staging of reproduction development in Cadenza wheat by non-destructive spike analysis
Source: J Exp Bot. 2020 Apr 7;71(12):3475–84. doi: 10.1093/jxb/eraa156 (PMC7307855; doi:10.1093/jxb/eraa156)
Supplement: eraa156_suppl_supplementary_table_S1_figure_S1 [file eraa156_suppl_supplementary_table_s1_figure_s1.pdf]

a

	Rice	Barley	Wheat.A	Wheat.B	Wheat.D
AtMS1 AT5G22260	54% BGIOSGA030870	86% HORVU5Hr1G067420	96.7% TraesCS5A02G233600	96.9% TraesCS5B02G232100	97.1% TraesCS5D02G240500
AtMYB26 AT3G13890	--	52% HORVU3Hr1G066860	93.6% TraesCS3A02G251200	95.8% TraesCS3B02G280700	94.8% TraesCS3D02G251600
AtDYT1 AT4G21330	47% Os07g0549600	83% HORVU2Hr1G042710	97.9% TraesCS2A02G212200	97.1% TraesCS2B02G237300	96.7% TraesCS2D02G218100

b

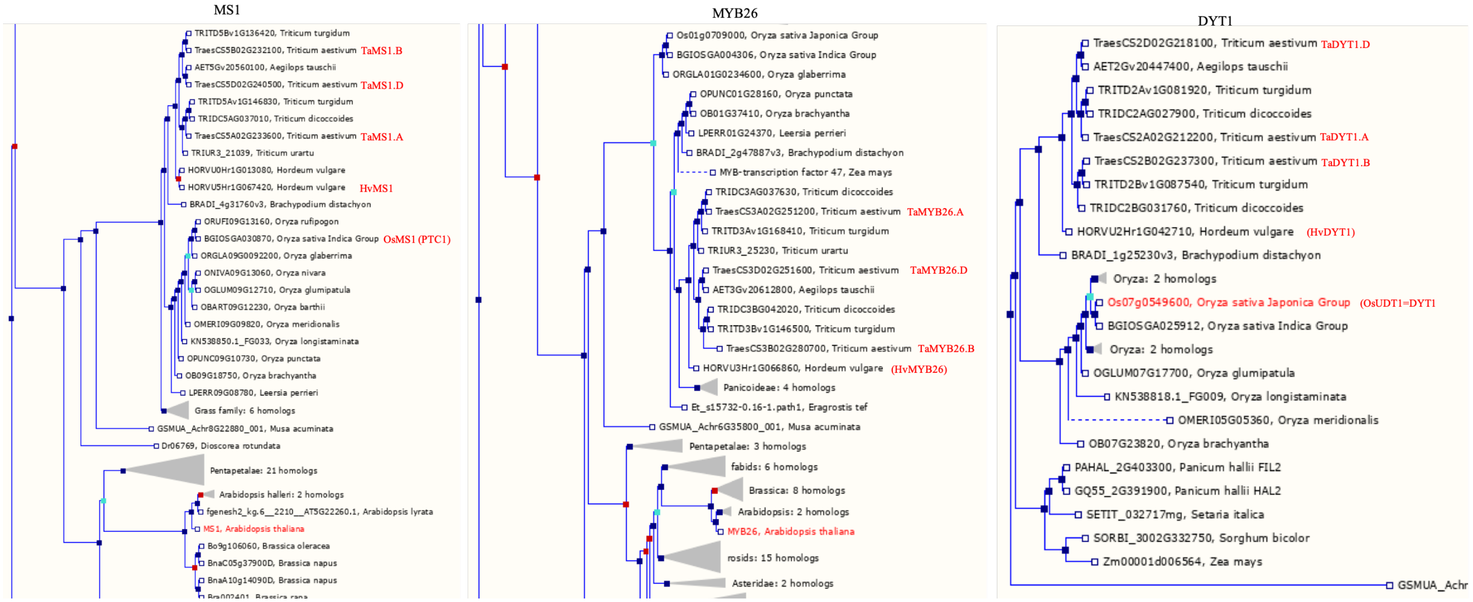

Supplementary Figure 1. Inter-species similarities between the three anther transcription factors used for expression analysis. a) NCBI Blast (https://blast.ncbi.nlm.nih.gov/Blast.cgi) was used for alignment between the Arabidopsis-Rice and barley, whilst EnsemblePlants was used to find the similarities between the barley orthologue and wheat homologues. b) Phylogenetic trees generated for the transcription factors.

	Forward	Reverse
TaActin	CCTCTCTGCGCCAATCGT	TCAGCCGAGCGGGAAATTGT
TaMS1	GTGCTGTTCCGGTTCGACTC	CAAGGCTCACCGTCCTTACG
TaMYB26	AGGGTGCTAGGGAACAGGTG	GAGGAAGGGATGTTACCGGAGT
TaDYT1	GTCGTGCCCAACATCACAAA	CACCTGACCCTGATAATGGA

Supplementary Table 1. Primers used for qRT-PCR analysis of wheat anther-specific transcription factors.
